# Supplementary material for: Identification of photoperiod-induced specific miRNAs in the adrenal glands of Sunite sheep (Ovis aries)
Source: Front Vet Sci. 2022 Jul 22;9:888207. doi: 10.3389/fvets.2022.888207 (PMC9354845; doi:10.3389/fvets.2022.888207)
Supplement: Supplementary file 1 [file Data_Sheet_1.ZIP › Supplementary file/miRDeep2 file.zip!/miRDeep2 file/Novel_Structure/SPLP42A3/Novel_26.pdf]

Provisional ID	:	NC_019469.2_23023
Score total	:	7.2
Score for star read(s)	:	3.9
Score for read counts	:	0.6
Score for mfe	:	1.1
Score for randfold	:	1.6
Score for cons. seed	:	
Total read count	:	13
Mature read count	:	12
Loop read count	:	0
Star read count	:	1

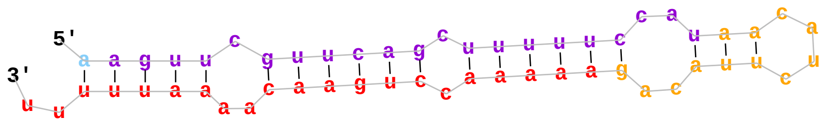

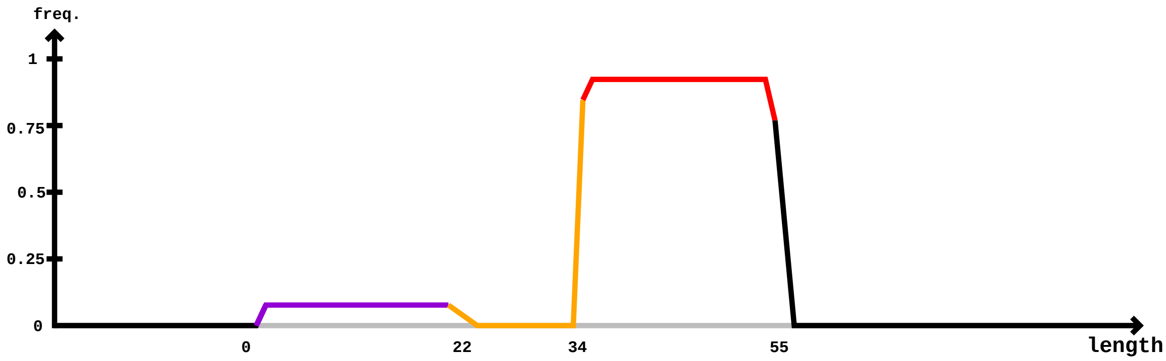

Star **Mature**

```
5'- uacuacuggguuggccaaaaaguucguucagcuuuuuccauaacaucuuacagaaaaaccugaacaaaauuuuuuugccaacccaacauaaagaaaacccuaaagacucua   -3'    obs
    uacuacuggguuggccaaaaaguucguucagcuuuuuccauaacaucuuacagaaaaaccugaacaaaauuuuuuugccaacccaacauaaagaaaacccuaaagacucua          exp
    ......(((((((((.((((((((.((((((.((((((..(((....)))..)))))).))))))..)))))))).))))))))).........................    reads     mm        sample
    ....................aguucguucagcuuuuuccau......................................................................    1         0         seq
    .....................................................aaaaaccugaacaaaCuuuu......................................    2         1         seq
    .....................................................aaaaaccugaacaaaCuuuuu.....................................    7         1         seq
    .....................................................aaaaaccugaacaaaCuuuuuu....................................    2         1         seq
    ......................................................aaaaccugaacaaaCuuuuu.....................................    1         1         seq
```
